# Spider Transcriptomes Identify Ancient Large-Scale Gene Duplication Event Potentially Important in Silk Gland Evolution

**DOI:** 10.1093/gbe/evv110

**Published:** 2015-06-08

**Authors:** Thomas H. Clarke, Jessica E. Garb, Cheryl Y. Hayashi, Peter Arensburger, Nadia A. Ayoub

**Affiliations:** ^1^Department of Biology, Washington & Lee University; ^2^Department of Biological Sciences, University of Massachusetts, Lowell; ^3^Department of Biology, University of California, Riverside; ^4^Department of Biological Sciences, California State Polytechnic University, Pomona

**Keywords:** Theridiidae, *Latrodectus*, *Steatoda*, expression, patterns of selection, neofunctionalization

## Abstract

The evolution of specialized tissues with novel functions, such as the silk synthesizing glands in spiders, is likely an influential driver of adaptive success. Large-scale gene duplication events and subsequent paralog divergence are thought to be required for generating evolutionary novelty. Such an event has been proposed for spiders, but not tested. We de novo assembled transcriptomes from three cobweb weaving spider species. Based on phylogenetic analyses of gene families with representatives from each of the three species, we found numerous duplication events indicative of a whole genome or segmental duplication. We estimated the age of the gene duplications relative to several speciation events within spiders and arachnids and found that the duplications likely occurred after the divergence of scorpions (order Scorpionida) and spiders (order Araneae), but before the divergence of the spider suborders Mygalomorphae and Araneomorphae, near the evolutionary origin of spider silk glands. Transcripts that are expressed exclusively or primarily within black widow silk glands are more likely to have a paralog descended from the ancient duplication event and have elevated amino acid replacement rates compared with other transcripts. Thus, an ancient large-scale gene duplication event within the spider lineage was likely an important source of molecular novelty during the evolution of silk gland-specific expression. This duplication event may have provided genetic material for subsequent silk gland diversification in the true spiders (Araneomorphae).

## Introduction

Evolutionary novelty at the molecular level is often attributed to gene duplication events followed by divergent molecular evolution, allowing one of the gene copies (paralog) to gain a different function (Ohno 1970; Kaessmann 2010). Genome-wide studies of paralog pairs demonstrate that genetic innovation arises through changes in protein structure and gene regulation (e.g., Kassahn et al. 2009). Duplicated gene pairs are implicated in the evolution of new phenotypes in numerous taxa (Braasch et al. 2009; Sato et al. 2009; Chapman et al. 2012; Hoffmann et al. 2012). Large-scale duplication events, such as whole-genome duplications, generate vast quantities of paralog pairs that could evolve new functions (Ohno 1970; Kasahara 2007; Kassahn et al. 2009). These events, while most commonly studied in plants (e.g., Vision et al. 2000; Jiao et al. 2011; Albert et al. 2013), are also found throughout the eukaryote phylogeny as revealed by whole-genome sequencing projects (Langham et al. 2004; Kasahara 2007; Sémon and Wolfe 2008), and may be vital for the evolution of a novel tissue (e.g., Kasahara 2007; Soltis et al. 2007).

Spiders (order Araneae) represent an excellent test case for examining the role of duplicated genes in the evolution of novel tissue-specific functions implicated in adaptation and species radiation. The common ancestor of spiders evolved specialized abdominal glands for the production of silk approximately 400 Ma (Selden et al. 2008; Foelix 2010; Starrett et al. 2012). Following this innovation, spiders diversified into over 44,000 described species that are important predators in almost all terrestrial ecosystems (Foelix 2010; Platnick 2014). Modifications to silk gland morphology, web-architecture, and silk genes within spiders likely contributed to their adaptive success (Bond and Opell 1998; Blackledge et al. 2009; Garb et al. 2010).

Consistent with the hypothesis that gene duplication is important for the evolution of functional novelty, the primary protein components of silk fibers are encoded by members of a gene family called spidroins—spider fibrous proteins (Hinman and Lewis 1992; Guerette et al. 1996; Gatesy et al. 2001). The number of spidroin paralogs is positively correlated with the number of functionally distinct silk gland types (Garb et al. 2007, 2010; Starrett et al. 2012). For instance, members of the suborder Mygalomorphae (tarantulas and their kin) possess undifferentiated spherical-shaped silk glands and only two or three spidroin paralogs (Garb et al. 2007, 2010; Starrett et al. 2012). In contrast, within the suborder Araneomorphae (so called true spiders), the Araneoidea (a clade of orb web weaving spiders and their kin) possess seven morphologically differentiated silk gland types that each synthesizes a task-specific fiber or glue (Vollrath and Selden 2007; Foelix 2010). One or more unique spidroin paralogs are highly expressed in each of six of these gland types (e.g., Guerette et al. 1996; Garb et al. 2010; Lane et al. 2013).

The duplication events underlying the diversification of spidroins and other silk-expressed genes have been difficult to address due to the limited genomic resources available for spiders. Nevertheless, a whole genome or segmental duplication event has been proposed to have occurred within the spider lineage based on the presence of two copies of at least three of the *Hox* genes in the wandering spider, *Cupiennius salei*, but only one in most other arthropods (Schwager et al. 2007). More recently, a multitissue transcriptomic analysis of the Western black widow (*Latrodectus hesperus*) greatly expanded the potential number of genes involved in silk production: Over 600 transcripts uniquely or significantly more expressed in silk glands than other tissues were identified, which we refer to as silk-specific transcripts (SSTs) (Clarke et al. 2014), many of which appear to be members of gene families.

Here, we use a comparative transcriptomics approach to examine the role of gene duplications in the evolution of silk gland-specific gene expression. Even though transcriptomes lack information about gene arrangement, which is typical evidence for whole-genome duplications, phylogenetic and molecular distance analyses of thousands of gene families can be used to test for a concentration of duplication events to a specific point in a phylogeny. The presence of multiple paralog pairs of similar age is strong evidence of a large-scale duplication event (e.g., Wolfe and Shields 1997; Vision et al. 2000; McKain et al. 2012). Transcriptomes de novo assembled from deep RNA-sequencing successfully identified whole-genome duplication events in the Agave lineage (McKain et al. 2012), and the raw sequencing reads used in transcriptome assembly provide estimates of tissue-specific transcript abundance.

We generated multitissue transcriptomes for three closely related cobweb weaving spider species, the Western black widow *L. hesperus*, the brown widow *Latrodectus geometricus*, and the false black widow *Steatoda grossa* (Theridiidae). We chose these species to validate tissue-specific gene expression patterns found in the black widow, but the choice of three close relatives also facilitated analyses of paralogs based on de novo assembled transcriptomes. Specifically, homologous transcripts found in all three species are likely correctly assembled and are likely to encode proteins. In addition, using close relatives permitted high-confidence paralogy assignments as there is less scope for reciprocal gene loss compared with more distantly related species. We then compared our transcriptomes with recently published arachnid genomes or transcriptomes spanning the phylogenetic range in which a large-scale duplication event was expected to occur to determine the relative phylogenetic placement of duplication events occurring in different gene families. We also examined the role of large-scale and/or isolated gene duplication events in the evolution of silk-specific gene expression in spiders. We hypothesize that a duplication event would be more likely to retain both paralogs if at least one is an SST compared with if both are generally expressed, because tissue-specific expression is a typical correlate of paralog retention (Adams et al. 2003; Huerta-Cepas et al. 2011). If true, we further predict that SSTs will have higher rates of amino acid replacements because paralogous gene pairs evolve, on average, under relaxed purifying selection compared with genes without paralogs (as in Mano and Innan 2008; Yang and Gaut 2011; Singh et al. 2013). We tested these predictions by comparing duplication and sequence evolution patterns between gene families with and without SSTs.

## Materials and Methods

### cDNA Libraries and Sequencing

Adult *L. hesperus* were collected in Riverside (Riverside County, CA) in March 2009, July 2010, and August 2011. Adult *L. geometricus* were collected in San Diego (San Diego County, CA) in July 2010 and Riverside County in August 2011. Adult *S. grossa* were obtained from SpiderPharm (Yarnell, AZ) in July 2010 and August 2011. For each species, 12 different tissue types were dissected from approximately 30 adult females (supplementary table S1, Supplementary Material online). Total RNA was isolated by homogenizing tissue derived from up to 29 individuals (supplementary table S1, Supplementary Material online) in TRIzol (Invitrogen) and was further purified with the RNeasy kit (Qiagen). Potentially contaminating DNA was removed with Turbo DNase (Ambion).

Seven cDNA libraries were prepared with the mRNA Sequencing Sample Preparation Kit (Illumina, San Diego, CA) followed by paired-end sequencing in single lanes of the Genome Analyzer I or II (Illumina) as described in Clarke et al. (2014). Remaining cDNA libraries were generated with the TruSeq RNA Sample Preparation Kit (Illumina) by Johns Hopkins Deep Sequencing and Microarray Core Facility (supplementary table S1, Supplementary Material online). Libraries were individually barcoded and pooled for paired-end sequencing of up to eight libraries per lane of the HiSeq 2000 at the UC Riverside Institute for Integrative Genome Biology Core Facility. The reads were cleaned with the RNA-Clean program (https://github.com/arensburger/RNAseq-clean, last accessed June 19, 2015) by removing barcodes, low quality reads, and sequences with BOWTIE (Langmead and Salzberg 2012) matches to known arthropod ribosomal RNA.

### Assembly of the Transcriptomes

**Fig. 1.—:**
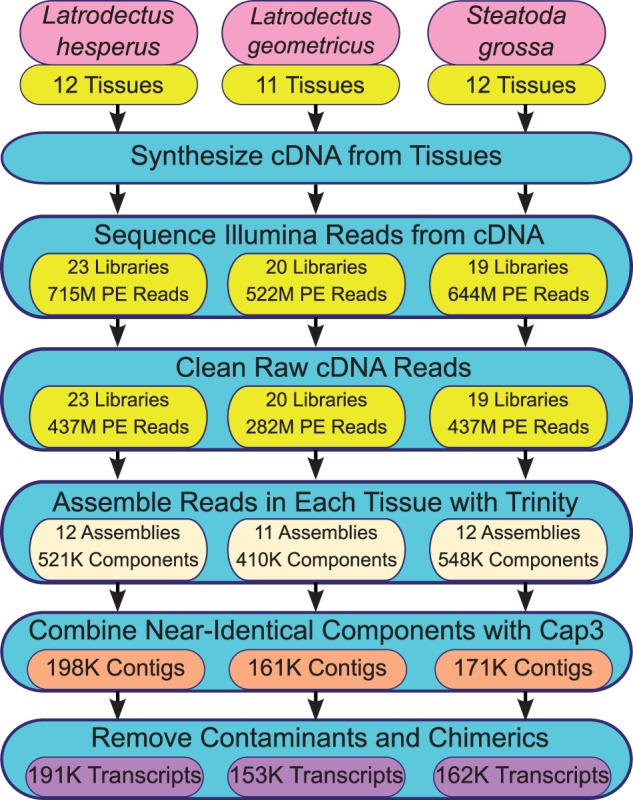
Flowchart detailing assembly of multitissue transcriptomes for three species of cobweb weaving spiders. Using RNA collected from multiple tissues (supplementary table S1, Supplementary Material online), cDNA was synthesized, sequenced, and cleaned of low quality and rRNA reads (yellow buttons). Each tissue-specific library was then subject to de novo assembly with Trinity (beige buttons) and these assemblies were combined to create a draft transcriptome (orange buttons). Probable contaminants and chimerics were removed from the draft to obtain the final transcriptomes (purple buttons).

Illumina sequence reads were assembled into species-specific transcriptomes using a multistep process (fig. 1). High-quality, nonribosomal RNA reads derived from the same species and tissue were combined and initial transcripts assembled using Trinity with default settings (Grabherr et al. 2011). Trinity produces multiple transcripts (usually interpreted as isoforms) that belong to the same component (interpreted as genes). Only the longest transcript for each component was retained for combination of the tissue-specific assemblies to remove potential isoforms. Tissue-specific assemblies were combined into a single draft transcriptome for each species by comparing the Trinity transcripts using BLAT (Kent 2002), clustering those that matched each other along the boundaries of each transcript by at least 98% identity over the full length of the match (of at least 50 bp). The 98% identity was chosen as the cutoff to allow for mismatches that might arise through sequencing errors. CAP3 was used to generate contiguous sequences (contigs) for each cluster (Huang and Madan 1999). CAP3 did not always combine all the sequences in the cluster. We retained contigs and singletons from each CAP3 assembly for our draft transcriptome.

The draft transcriptome was pruned of possible contaminants and chimerics. All contigs that had a best BLASTX (Altschul et al. 1990) hit in UniProt to a bacterial sequence with an *e* value less than 1e-50 were flagged as bacterial contaminants and removed. For a more detailed description of the identity of the bacterial sequences, refer to supplementary figure S3, Supplementary Material online. Probable chimeric assembly errors were identified using a Python script (Clarke et al. 2014), based on BLASTX comparisons between the transcriptomes and the *Drosophila melanogaster* translated genes (version 5.50 from FlyBase) and removed. Potential cross-species contaminants generated during cDNA library construction were identified by comparing the draft transcriptomes of all three species using BLASTCLUST with parameters of 99% length coverage and 99% nucleotide identity (ftp://ftp.ncbi.nih.gov/blast/documents/blastclust.html, last accessed June 19, 2015). Raw high quality sequence reads were aligned to these near-identical contigs using BOWTIE in a species-specific manner and abundance estimated using RSEM (Li and Dewey 2011). Estimated read counts per million total reads (RSEM’s TPM) were summed across the tissue-specific libraries within each species. To prevent removal of very slowly evolving homologs that are actively transcribed, only those contigs with summed TPM less than 1% of the most abundant contig in the BLASTCLUST cluster were identified as cross-species contaminants and removed. We refer to the set of contigs pruned of contaminants as the final transcriptome, and consider this set to contain high-quality transcripts, hereafter referred to as transcripts (see fig. 1).

### Annotation

The final transcriptomes were annotated using BLASTX determined homology at an *e*-value cutoff of 1e-5 to UniProt/Trembl (obtained September 18, 2013), the *D. melanogaster* translated genes (v 5.50, obtained from FlyBase), and the deer tick, *Ixodes scapularis*, translated genes (v 1.2 obtained from VectorBase). The transcripts were translated into amino acid sequences using the longest open reading frame (ORF), either in the same frame as the best UniProt hit or in all frames for the transcripts with no BLAST-based homology to UniProt proteins. Amino acid sequences that were bounded by stop codons at both the 5′- and 3′-end and for which the length of the protein after the first methionine (M) was at least 75% of the total length were trimmed to that first M.

The completeness of the transcriptomes was ascertained by comparison to a set of eukaryotic proteins using CEGMA (Parra et al. 2007) and to single copy orthologs identified in 30 arthropod species (BUSCO as described in Waterhouse et al. 2013). For the BUSCO data set, the completeness was quantified using the BUSCO provided Perl scripts. In brief, the scripts first estimate the number of genes in a benchmark species found in the BUSCO data set, here the deer tick translated genes v1.2 (which is the most closely related species to spiders represented in BUSCO). Second, the BUSCO scripts compare the number of homologous genes in the test transcriptomes with the number of homologous genes that the benchmark species has in the BUSCO data set (further details available at ftp://cegg.unige.ch/OrthoDB7/BUSCO/README.txt, last accessed June 19, 2015).

### Generation of Gene Clusters in *Latrodectus*, *Steatoda*, and Other Arachnids

**Fig. 2.—:**
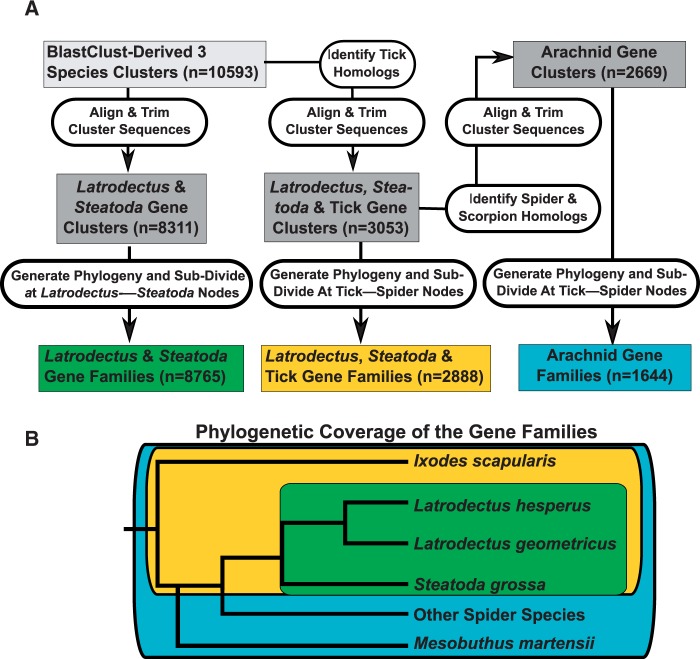
Delineation of gene families. (*A*) Transcripts derived from our three cobweb weaving species were grouped with BLASTCLUST to identify clusters of homologous genes (see text for complete methods). Those with a representative member from all three species formed the basis for defining gene clusters (dark gray boxes). Either no homologs were added and sequences were aligned and trimmed to form *Latrodectus* & *Steatoda* gene clusters, or homologous tick sequences were added, aligned, and trimmed to form *Latrodectus, Steatoda* & tick gene clusters. In addition, homologous sequences from seven other published spider and one scorpion gene sets were added to *Latrodectus, Steatoda* & tick gene clusters to form arachnid gene clusters. Each cluster was subject to phylogenetic analyses and putative gene families delineated (colored boxes) by dividing gene trees at either the *Latrodectus–Steatoda* speciation node (*Latrodectus* & *Steatoda* gene families) or at the tick–spider speciation node (*Latrodectus, Steatoda* & tick gene families and arachnid gene families) as per the species phylogeny in (*B*).

We used a multistep approach to cluster genes with three levels of taxonomic inclusion followed by phylogenetic analyses to delineate putative gene families (see fig. 2). We used our three cobweb weaver transcriptomes as the core of the clustering, with the requirement that each cluster must contain representative sequences from all three species for two reasons. First, de novo assembled transcriptomes could include incorrectly assembled transcripts or nonprotein-coding regions of DNA (due to the depth of sequencing). By requiring sequence homology among all three species we had confidence that the transcripts were correctly assembled and were likely protein coding. Even though we required representatives of all three species, we should still capture a high proportion of within species paralogs due to the fact that the three species are closely related and will have been unlikely to have differentially lost a significant number of paralogs. Second, requiring the inclusion of *L. hesperus* transcripts allowed functional annotation of the gene family in terms of involvement with silk production (Clarke et al. 2014).

**Fig. 3.—:**
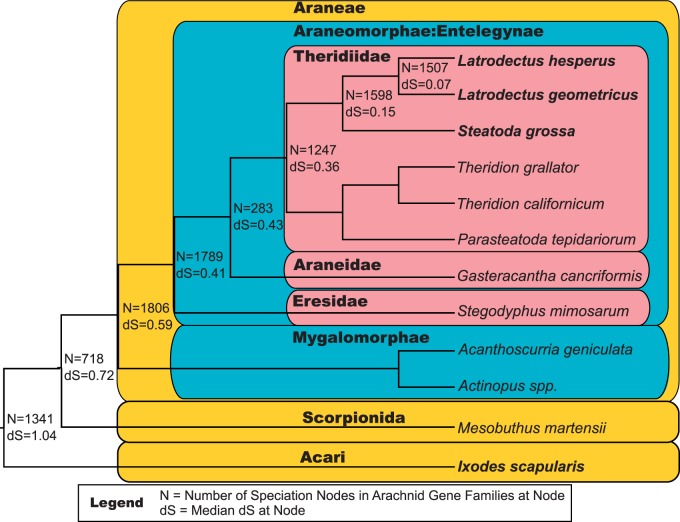
Molecular distances of arachnid speciation events. The species tree is based on Bond et al. (2014), Coddington (2005), and Fernández et al. (2014) for relationships among spider suborders and families, Arnedo et al. (2004) for relationships within theridiids, and Regier et al. (2010) for relationships among arachnid orders. The median d*S* (number of synonymous substitutions per synonymous sites) for all speciation nodes (*N* = number of nodes) identified in arachnid gene families is shown above branches (see fig. 2 and Materials and Methods for delineation of gene families).

Although our three cobweb weaver transcriptomes ensured a high quality set of gene clusters, these three species diverged well after the large-scale duplication event suggested by the *Hox* genes (Schwager et al. 2007). Thus, we generated two additional sets of gene clusters that incorporated homologs from other arachnid species in order to place our theridiid duplicates in a phylogenetic context. The first added homologs from the deer tick, *I. scapularis,* which has the best characterized and annotated genome of any sequenced arachnid. The second set incorporated homologs from an additional eight arachnid species with whole genomes or deeply sequenced transcriptomes (figs. 2 and 3). These additional species included two with complete genomes: The spider species *Stegodyphus mimosarum* (Sanggaard et al. 2014) and a nonspider arachnid, the scorpion *Mesobuthus martensii* (Cao et al. 2013). Another six spider species, for which only transcript data were available, were also added from National Center for Biotechnology Information (NCBI)’s dbEST (*Parasteatoda tepidariorum,* obtained November 14, 2013), NCBI’s Transcript Shotgun Assembly (*Theridion grallator* (PRJNA217184), and *Theridion californicum* (PRJNA217181), both obtained November 14, 2013), Prosdocimi et al. (2011) (*Actinopus* sp*.* and *Gasteracantha cancriformis*), and Sanggaard et al. (2014) (*Acanthoscurria geniculata*). Transcripts within each of the first three transcriptomes were combined using CAP3 to mirror our own transcriptomes; other transcriptomes had been subject to CAP3 prior to publication. The transcripts from the first five transcriptomes were translated by finding the longest ORF in the same frame as the alignment with the cobweb weaver transcriptomes with the lowest *e* value as determined by BLASTN. We used the translated transcripts published by Sanggaard et al (2014) for *A. geniculata.*

The three sets of gene clusters were generated consecutively with each prior gene cluster serving as the seed for the next gene cluster (fig. 2*A*). First, *L. hesperus*, *L. geometricus*, and *S. grossa* translated transcripts were grouped using BLASTCLUST with parameters of 60% length coverage and 70% amino acid identity. These parameters maximized the number of transcripts included in clusters, while minimizing inclusion of nonhomologous transcripts into the same cluster (supplementary fig. S1, Supplementary Material online). The BLASTCLUST-derived clusters were trimmed and very short sequences removed (detailed below). After trimming, clusters that still contained a representative from each of our three species were designated *Latrodectus* & *Steatoda* gene clusters. Second, tick homologs were added to the BLASTCLUST-derived clusters based on BLASTX alignments; a tick protein sequence was considered homologous if it had a best BLASTX alignment to each of the three species in the same BLASTCLUST-derived cluster, provided that the BLASTX alignment had an *e* value of less than 1e-5. Clusters containing at least one representative from each spider and tick species were subject to multiple alignments with MUSCLE and very short sequences, which could represent incompletely assembled transcripts or potentially shorter isoforms, were removed (described in detail below) to generate the *Latrodectus*, *Steatoda* & tick gene clusters. Third, other spider and scorpion homologs were added to the *Latrodectus*, *Steatoda* & tick gene clusters using a similar process to the addition of tick homologs to BLASTCLUST-derived clusters, except that BLASTN was used to identify homologs between the other arachnid genes or transcripts and the transcripts in the *Latrodectus*, *Steatoda* & tick gene clusters. Those clusters that contained at least one species in addition to *L. hesperus, L. geometricus*, *S. grossa*,** and tick after alignment and removal of short sequences (described in detail below) formed the arachnid gene clusters (fig. 2*A*).

As many of our species’ transcripts were derived from de novo assembled transcriptomes, there were a number of partial sequences that could bias alignments and phylogenetic analyses. To remove these very short incomplete sequences from the final gene clusters, the amino acid sequences within each initial set of clusters were aligned using MUSCLE with default settings (Edgar 2004). The alignments were trimmed to the boundaries of the shortest out of the longest amino acid sequences from each species. Amino acid sequences with gaps in greater than 25% of the sites of the trimmed alignment were removed. The 25% cutoff allowed some partial sequences to be retained to ensure the greatest taxonomic sampling possible, but allowed sufficient depth of coverage to calculate molecular distances.

### Delineation of Gene Families by Phylogenetic Analyses

Putative gene families were identified from the gene clusters by calculating gene trees and identifying clades of sequences that derived from the *Latrodectus–Steatoda* divergence or the spider–tick divergence (fig. 2). First, amino acid sequences in each gene cluster were realigned using MUSCLE with default parameters as the prior alignments might have been biased by short sequences. Poorly aligned columns were removed using trimAl under the “strictplus” setting (Capella-Gutiérrez et al. 2009), which optimizes the signal-to-noise ratio in the alignments, but retains columns with identical amino acids. Codon alignments were constructed by replacing the amino acid sequences with the underlying nucleotide sequences with a custom Perl script. These high-quality codon alignments were used to infer gene trees with TreeBeST (http://treesoft.sourceforge.net/treebest.shtml, last accessed June 19, 2015), which was found to assign gene duplication events consistent with the biological expectation for duplications to be rare and with syntenic evidence for duplications more often than gene tree reconstruction methods based solely on sequence data followed by species tree reconciliation (Vilella et al. 2009) and this program has been used in multiple phylogenomic studies (e.g., Dickerson and Robertson 2011; Boussau et al. 2013; Ge et al. 2013). TreeBeST searches for an optimal gene tree given a species tree, by first inferring multiple gene trees, including trees derived from neighbor-joining and PhyML under multiple models of evolution, as well as trees that make no use of the sequence information, but simply minimize gene duplications and losses based on a user-supplied species tree (we used the species trees in figs. 2*B* and 3). TreeBeST then produces a final rooted tree based on an optimal tree merging algorithm (Cocke–Kasami–Younger top-down algorithm), weighing possible gene trees both on the bootstrap support values from neighbor-joining and PhyML trees and the number of duplications and losses in the tree.

We ensured that all duplication nodes in each TreeBeST gene tree occurred after the divergence of spiders and ticks by focusing on clades rooted at the spider–tick speciation node. If necessary, *Latrodectus*, *Steatoda* & tick gene clusters and arachnid gene clusters were subdivided by splitting the gene trees at the spider–tick speciation node with a custom Perl script. We retained only the clades containing all four species, resulting in the *Latrodectus*, *Steatoda* & tick gene families, or containing all four species and an additional species resulting in the arachnid gene families (fig. 2). The *Latrodectus* & *Steatoda* gene clusters were not subdivided when searching for duplication nodes, as the suspected large-scale duplication event is supposed to predate this divergence (Schwager et al. 2007). However, the *Latrodectus* & *Steatoda* gene clusters were subdivided at the *Latrodectus–Steatoda* speciation node to generate *Latrodectus* & *Steatoda* gene families for analysis of patterns of selection.

### Identification and Phylogenetic Placement of Spider-Specific Gene Duplications

Within each TreeBeST gene tree, duplication and speciation nodes were identified. Relative molecular distances for duplication and speciation events were estimated from the number of synonymous substitutions per synonymous site (d*S*) using PAML with the M0 model (Yang 2007) using the TreeBeST trees for *Latrodectus* & *Steatoda* gene clusters, *Latrodectus*, *Steatoda* & tick gene families, and arachnid gene families. The distance used for each duplication node was the mean pairwise d*S* value of all extant intraspecies paralogs derived from the duplication node to prevent highly divergent paralog pairs from dominating the molecular distance. Only duplication nodes with d*S* < 2 (to avoid saturation as found in Vanneste et al. 2013), and d*S* ≥ 0.01 (to avoid incorrectly inferring within-species allelic variants or isoforms as paralogs) were analyzed.

Duplication nodes belonging to an ancient whole genome or other large-scale duplication event should have similar molecular distances that fit a normal (Gaussian) distribution due to some variation in rates of evolution among gene families, as in Jaillon et al. (2007), McKain et al. (2012), and Vision et al. (2000). To determine whether arachnid duplication nodes conformed to this expectation, the mean d*S* distances were used to model Gaussian mixtures using the R-package mixtools (Benaglia et al. 2009). The returned posterior probability (PP) was used to assign duplication nodes to specific d*S* distributions with 95% as a cut-off.

In order to determine the phylogenetic position of duplication events, we also examined individual gene trees. Bayesian trees were estimated with MRBAYES using two chains with one run (Ronquist et al. 2012) for 1,000,000 generations or until the standard deviation of split frequencies was less than 0.015 for the arachnid gene families with a mygalomorph representative, a scorpion representative, and a paralog pair in one of our cobweb weaving species with a duplication node assigned to an older Gaussian distribution. Bayesian trees were rooted and reconciled with the species tree in figure 3 using Notung (Stolzer et al. 2012) to identify duplication and speciation nodes. Duplication nodes that were polytomies or restricted to a single species were not considered.

### Molecular Evolutionary Patterns of SSTs

We identified gene clusters and gene families containing transcripts primarily expressed in silk glands by comparing the newly assembled *L. hesperus* transcriptome to transcripts described in Clarke et al (2014) using BLASTN. A new *L. hesperus* transcript was considered silk specific if it had the best BLASTN alignment to an SST described in Clarke et al. (2014) with an *e* score < 1e-50. The same process was used to identify transcripts that had significant expression levels (expected counts per million [eCPM] ≥ 1 in silk glands, venom glands, or cephalothoraxes) but were not SSTs in Clarke et al. (2014), and those transcripts that did not meet the significant expression threshold (eCPM < 1). Gene families or clusters containing at least one SST were compared with gene families or clusters containing no SSTs but at least one significantly expressed transcript (non-SST) and to gene families or clusters without either (low expression) for size of gene family, dS of duplication nodes, and patterns of sequence evolution.

In order to infer patterns of selection on protein-coding sequences, we estimated a gene family-wide omega, which is the ratio of nonsynonymous substitutions per nonsynonymous site (d*N*) to synonymous substitutions per synonymous site (d*S*). Omega was calculated for each of the *Latrodectus* & *Steatoda* gene families and the *Latrodectus*, *Steatoda* & tick gene families with PAML using the M0 settings and the TreeBeST trees.

## Results

### Deep Sequencing of Multiple Tissues Generates Nearly Complete Transcriptomes for Three Spider Species

We generated high quality transcriptomes for three species of cobweb weaving spiders based on almost 2 billion sequence reads of genes expressed in 11–12 tissue types (fig. 1; supplementary table S1, Supplementary Material online). The resulting transcripts from each of the three species have similar length distributions for the nucleotide and amino acid sequences (supplementary fig. S2, Supplementary Material online). In each species, the transcripts show a slight bias to be 5′ incomplete (45%) rather than 3′ incomplete (30%) based on the absence of stop codons either 5′ or 3′ of the ORF (supplementary fig. S2, Supplementary Material online).

The high quality transcriptomes contain almost complete benchmarking gene sets including greater than 99% of the core eukaryotic genes (CEGs, > 95% complete) and greater than 96% of single copy arthropod orthologs (BUSCO, > 83% complete; table 1). The missing CEGs were shared between the two *Latrodectus* species. Furthermore, homologs of a majority of the tick (66%) and fruit fly (74%) protein-coding genes are in each of our three transcriptomes (table 1).

**Table 1:** Metrics of Completeness of the Three Cobweb Weaving Spider Species’ Transcriptomes

	Steatoda grossa	Latrodectus geometricus	Latrodectus hesperus
No. of fly genes^a^	9,928	9,915	10,176
No. of tick genes^b^	13,359	13,246	13,514
CEGMA, % complete^c^	97.6	95.6	99.6
CEGMA, % partial^c^	100	99.6	99.6
BUSCO, % complete^d^	90.5	83.4	93.1
BUSCO, % partial^d^	96.8	96.4	97.5

^a^Number of *Drosophila melanogaster* genes from the v5.50 translated gene set with a BLASTX *e* value<1e-5 compared with the transcriptome.

^b^Number of *Ixodes scapularis* genes from the v1.2 translated gene set with a BLASTX *e* value<1e-5 compared with the transcriptome.

^c^Reported complete and partial results from the CEGMA program.

^d^Reported complete and partial results using the BUSCO Perl scripts with the *I. scapularis* genes as a baseline.

BLASTCLUST of the three transcriptomes resulted in 10,593 clusters (containing 101,279 transcripts) with at least one transcript from each of our cobweb weaving spider species (supplementary fig. S4, Supplementary Material online). These clusters were much more likely to be annotated (92%) by BLAST-based homology (*e* value < 1-e5 of at least one member) to UniProt or one of two closely related species (fruit fly and deer tick) than were BLASTCLUST-derived clusters containing multiple transcripts, but not from all species (30,424 transcripts, 22% annotated) or singletons (330,172 transcripts, 10% annotated).

### Duplication Node d*S* Distribution Supports Ancient Large-Scale Duplication Event

**Fig. 4.—:**
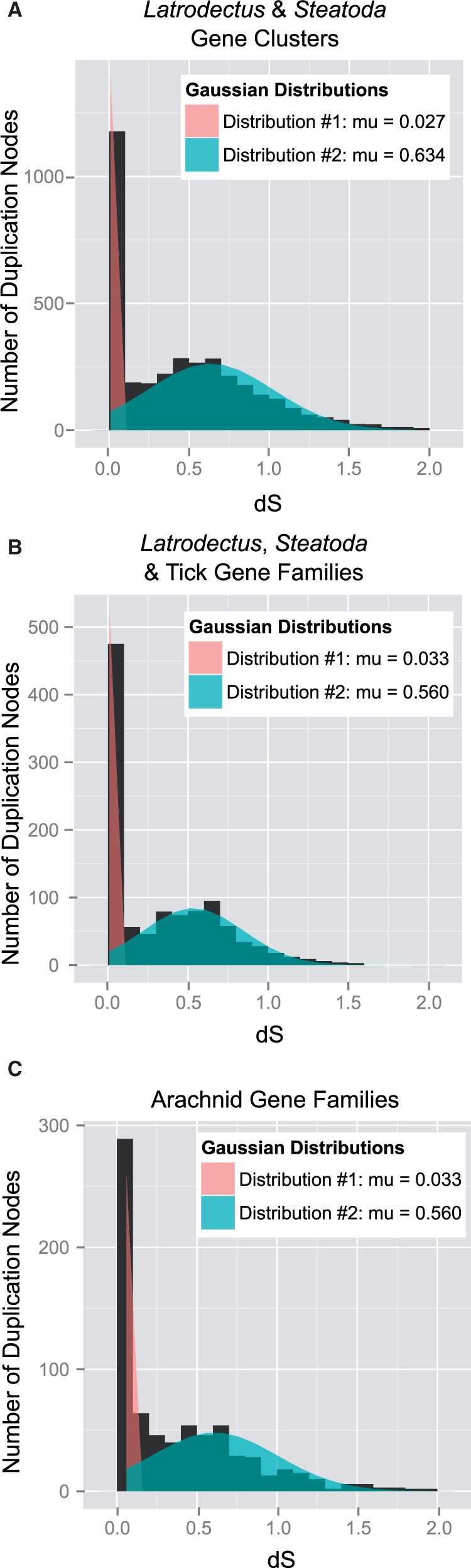
Molecular distances of duplication nodes support ancient large-scale duplication event. Distributions of the mean number of synonymous substitutions per synonymous site (d*S*) for paralogs descending from duplication nodes in groups of homologous genes with different taxonomic inclusion (see fig. 2 and Materials and Methods for delineation of gene clusters and families) show two peaks, one at d*S*∼0.05 and one at d*S*∼0.50. Gaussian mixture models support the presence of the two distributions (brown distribution, d*S*∼0.05; blue distribution, d*S*∼0.50). The older distribution (blue) is suggestive of an ancient large-scale duplication event.

Phylogenetic analyses of 8,311 *Latrodectus* & *Steatoda* gene clusters revealed 3,581 duplication nodes in 1,757 gene clusters (see fig. 2 for delineation of gene clusters). Focusing on putative gene families with an unambiguous tick homolog of similar length to each of the three spider species (2,888 *Latrodectus*, *Steatoda* & tick gene families) reduced the total number of duplication nodes to 1,086 in 681 gene families, but ensured that the duplication events happened after the divergence of spiders from ticks. Requiring a homolog from an additional arachnid species (1,644 arachnid gene families) further reduced the number of duplication nodes to 579 in 445 gene families, but increased the power to determine when duplication events happened in the arachnid phylogeny. The distributions of the mean d*S* of duplication nodes revealed two peaks in all sets, no matter the taxonomic inclusion: One peak with d*S* < 0.05, which probably represents recent duplications of individual genes, and a second peak at dS ∼ 0.50 with an observable valley between the two peaks (fig. 4). The second peak is not an artifact of rapidly evolving paralogs; within-species paralogous pairs that are each other’s closest relatives almost all have d*S* < 0.10 (see supplementary fig. S5, Supplementary Material online).

When d*S* values of duplication nodes were fit to two Gaussian distributions one was centered at d*S* ∼ 0.03 in all three sets of gene clusters or gene families (fig. 4). The average d*S* of the second distribution varied slightly among the sets of genes with μ = 0.63 for the *Latrodectus* & *Steatoda* gene clusters (fig. 4*A*), μ = 0.53 for the *Latrodectus*, *Steatoda* & tick gene families (fig. 4*B*), and μ = 0.56 for the arachnid gene families (fig. 4*C*). Adding more distributions further improved the likelihood of the Gaussian mixture models, but the means of these additional distributions were all young, d*S* < 0.08 (supplementary table S2, Supplementary Material online), indicating that the older distribution represents a single duplication event involving many genes. Indeed, a majority of duplication nodes were assigned to the older distribution in the *Latrodectus* & *Steatoda* gene clusters (69% of duplication nodes), the *Latrodectus*, *Steatoda* & tick gene families (58%), and the arachnid gene families (57%).

The older duplication distribution is also not a product of multiple duplication events in a few gene families: Approximately 60% of the *Latrodectus*, *Steatoda* & tick gene families that contain paralogs have a duplication node assigned to the older distribution (405, representing 14% of all the families). Furthermore, 79% of these 405 families have only a single duplication node assigned to the older distribution, again suggesting a single duplication of a large portion of the ancestral genome.

### Large-Scale Duplication Event Predates Spider Suborder Divergence

Comparison of the median d*S* of duplication nodes in arachnid gene families assigned to the older distribution (median d*S* = 0.52) to the median d*S* of eight arachnid speciation events (fig. 3) places the putative large-scale duplication event around the same time as the divergence of the spider suborders, Araneomorphae and Mygalomorphae (median d*S* = 0.59). The median d*S* of duplication nodes also suggests that the duplication event happened well before the divergence of the basal entelegyne, Eresidae from other entelegyne species (median d*S* = 0.41), and well after the divergence of spiders from scorpions (median d*S* = 0.72; fig. 3).

**Fig. 5.—:**
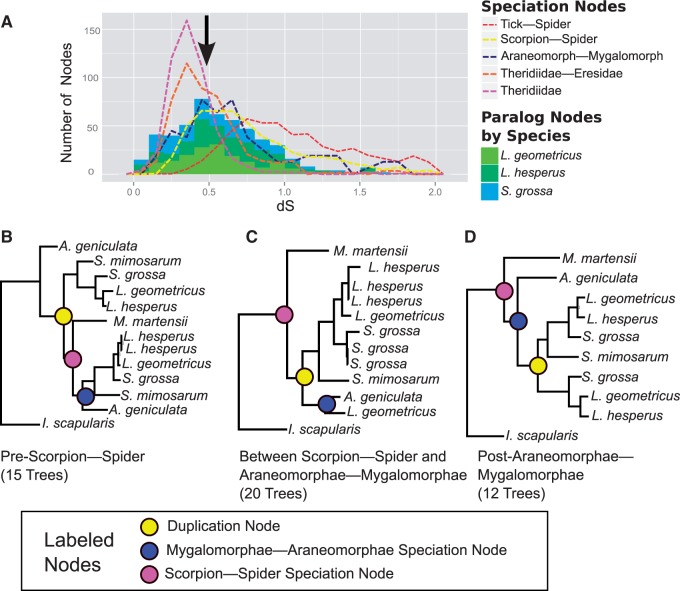
Molecular distances and gene trees support large-scale gene duplication event prior to spider suborder divergence. (*A*) The distribution of molecular distances (d*S*) in arachnid gene families (see fig. 2 and Materials and Methods for delineation of gene families) for duplication nodes is similar to the d*S* distribution for the Araneomorphae–Mygalomorphae divergence (blue-dotted line) and the spider–scorpion divergence (yellow-dotted line). Bars represent duplication nodes retaining paralog pairs in at least one of our three focal species that were assigned to the older Gaussian distribution (blue in fig. 4*C*). d*S* = mean number of synonymous substitutions per synonymous sites for paralogs descending from the duplication node or orthologs descending from speciation node. The d*S* distributions for speciation nodes were normalized to their total number and then scaled to the heights of the paralog distributions to ease the comparisons between the paralogs and speciation ages. (*B–D*) Exemplar gene trees showing phylogenetic placement of duplication events that predate the divergence of our cobweb weaving species. Bayesian gene trees that have a mygalomorph homolog, a scorpion homolog, and only one duplication node assigned to the older Gaussian distribution (blue in fig. 4*C*) reveal that most (20 trees) duplication events (yellow dots) are older than the Araneomorphae–Mygalomorphae divergence (blue dots) but younger than the spider–scorpion divergence (purple dots) (*C*). However, in 15 such trees the duplication node is older than the scorpion–spider divergence (*B*), whereas in 12 the duplication event is younger than the Araneomorphae–Mygalomorphae divergence (*D*).

As the median d*S* values give only single data points, we also compared the d*S* distributions between the duplication nodes and the speciation nodes in the arachnid gene families. The peak of the older duplication distribution in the arachnid gene families (black arrow in fig. 5*A*) is between the peaks of the distributions of the Mygalomorphae–Araneomorphae speciation nodes (blue-dotted lines in fig. 5*A*) and the scorpion–spider speciation nodes (yellow-dotted lines in fig. 5*A*). Additionally, the older d*S* duplication distribution clearly predates the radiation of theridiid genera (purple-dotted line) or the divergence between Eresidae and Theridiidae (orange-dotted line), but postdates the tick–spider speciation nodes (red-dotted line).

Comparisons of d*S* for duplication nodes to speciation nodes may not accurately reflect the timing of duplication events because of biased evolution among individual gene families, such as an increased rate of evolution for paralogs compared with single copy genes (Yang and Gaut 2011; Singh et al. 2013). Bayesian phylogenetic trees for the 106 arachnid gene families with a mygalomorph homolog, a scorpion homolog, and a paralog pair with a duplication node assigned to the older duplication peak tended to have low support for determining relative branching order of speciation and duplication nodes. Only 60 relevant nodes had PP ≥ 95% whereas 163 had PP ≥ 50% (supplementary table S3, Supplementary Material online). For the duplication and speciation nodes supported by PP ≥ 50%, 54 duplications occurred after the mygalomorph–araneomorph divergence, 68 before the divergence of scorpions and spiders, and the remaining 41 between the two divergence events. However, for trees with only one duplication event (exemplar trees in fig. 5*B* and *C*), a plurality supports the duplication occurring between the two divergence events (20 at PP ≥ 50%, 3 at PP ≥ 95%) rather than before the scorpion–spider divergence (15 at PP ≥ 50%, 0 at PP ≥ 95%) or after the mygalomorph–araneomorph divergence (12 at PP ≥ 50%, 3 at PP ≥ 95%).

### The Older Duplication Event Preferentially Enriched the Silk Gland Transcriptome

**Fig. 6.—:**
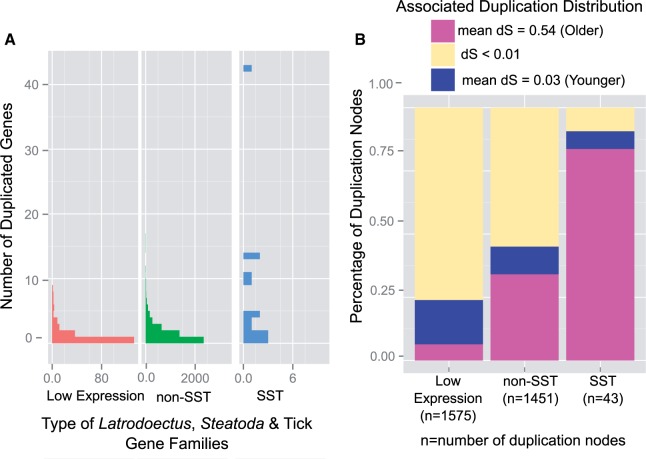
Gene families with SSTs have more members and are more likely to retain old paralogs than other gene families. (*A*) Within *Latrodectus, Steatoda* & tick gene families (see fig. 2 and Materials and Methods for delineation of gene families), those with SSTs are more likely to have a paralog (number of gene families with no paralogs are shown at the bottom) and also on average have more paralogs compared with gene families with non-SSTs or gene families with low expression transcripts (see Materials and Methods for identification of the three expression categories of gene families). (*B*) The duplication nodes in the SST-containing *Latrodectus, Steatoda* & tick gene families are almost all assigned to the older Gaussian distribution (blue in fig. 4*B*, pink bar here), and only a few are assigned to the younger Gaussian distribution (brown in fig. 4*B*, blue bar here) or have an d*S* < 0.01 (yellow bar). In contrast, duplication nodes from the *Latrodectus, Steatoda* & tick gene families that do not contain SSTs are more likely to have an d*S* < 0.03. d*S* = mean number of synonymous substitutions per synonymous sites for paralogs descending from the duplication node.

Consistent with prior findings that paralog retention is a correlate of tissue-specific expression (Adams et al. 2003; Huerta-Cepas et al. 2011), we found that genes predominately expressed in the silk glands of the black widow (SSTs from Clarke et al. 2014) are members of larger gene families than those that lack an SST. The *Latrodectus*, *Steatoda* & tick gene families that contained an SST had a median of 4.5 transcripts in addition to the base 4 (one transcript from tick, *L. hesperus*, *L. geometricus*, and *S. grossa* each) compared with a median of one additional transcript for the non-SST *Latrodectus*, *Steatoda* & tick gene families (Kruskal–Wallis test statistic = 18.85, *P* < 2e-5; fig. 6*A*). This difference is less pronounced for the number of duplication nodes (median duplication nodes = 3.5 in SST-containing *Latrodectus*, *Steatoda* & tick gene families, median duplication nodes = 1 for *Latrodectus*, *Steatoda* & tick non-SST gene families). In addition, the SST-containing *Latrodectus*, *Steatoda* & tick gene families are more likely to have divergent paralog pairs (mean d*S* = 0.27) than the non-SST *Latrodectus*, *Steatoda* & tick gene families (mean d*S* = 0.12; two sample *t*-test, *t* = 4.88, *P* = 3e-6). Furthermore, *Latrodectus*, *Steatoda* & tick gene family duplication nodes with SST descendants are assigned to the older peak (83%) at a more than 2-fold higher rate than duplication nodes whose descendants are all non-SSTs (34%) (fig. 6*B*).

### Silk Gland-Specific Transcripts Experienced Relaxed Selection

**Fig. 7.—:**
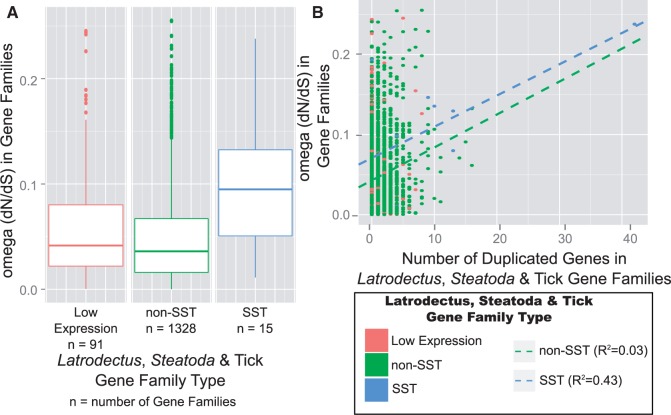
Gene families with SSTs have higher amino acid replacement rates than other gene families. (*A*) *Latrodectus, Steatoda* & tick gene families (see fig. 2 and Materials and Methods for delineation of gene families) containing SSTs (blue) have significantly higher omega (d*N*/d*S*) values than do those families containing non-SSTs (green) or only transcripts with low expression (red). See Materials and Methods for identification of the three expression categories of gene families. (*B*) There is a statistically significant positive correlation between gene family size and omega (dotted lines show best fit relationship), but the higher intercept value for the SST (blue-dotted line) compared with the non-SST (green-dotted line) containing gene families suggests unique selective pressures on transcripts specifically expressed in silk glands.

*Latrodectus*, *Steatoda* & tick gene families containing an SST had an average omega value (mean = 0.10) about twice that of non-SST *Latrodectus*, *Steatoda* & tick gene families (mean = 0.05, two sample *t*-test, *t* = 3.00, *P* < 0.009) (fig. 7*A*). This results from the increased d*N* values in SST-containing families (total d*N* tree length = 1.33 in SST families vs. 0.43 in non-SST families; two sample *t*-test, *t* = 3.00, *P* < 0.009), with little difference in the d*S* (total d*S* tree length = 14.0 in SST families vs. 17.0 in non-SST families; *t* = 1.75, *P* = 0.07), indicating that the increase in omega is primarily driven by a higher rate of amino acid replacements in the gene families with SSTs than ones without. Focusing on the more recently diverged sequences found in the *Latrodectus* & *Steatoda* gene families revealed slightly higher omega values on average, but SST-containing families still have higher omega values (mean = 0.15) than non-SST families (mean = 0.07; two sample *t*-test, *t* = 7.53, *P* < 1e-10).

**Fig. 8.—:**
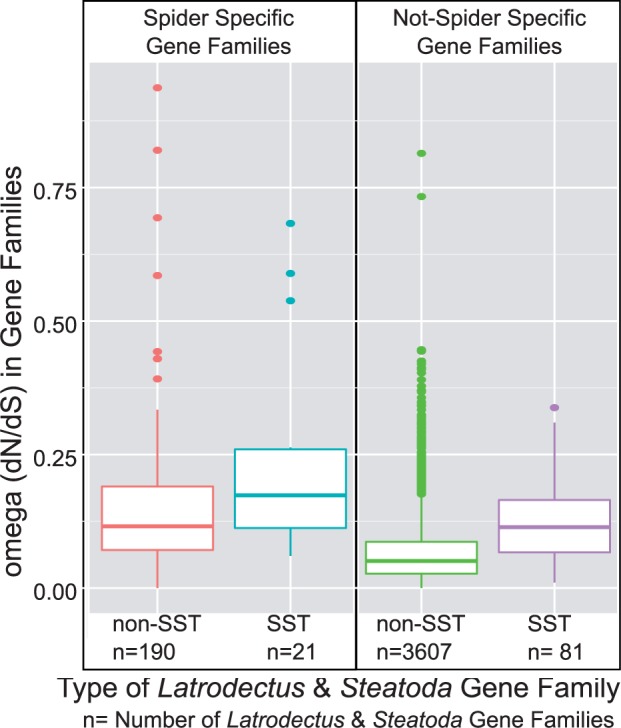
Spider lineage-specific gene families have high amino acid replacement rates. In *Latrodectus* & *Steatoda* gene families (see fig. 2 and Materials and Methods for delineation of gene families), the omega (d*N*/d*S*) values are higher for families without homologs outside of spiders (Spider Specific) than those families with nonspider homologs (Not Spider Specific). Within either category, SST-containing gene families have higher omega than non-SST-containing gene families. See Materials and Methods for identification of the expression categories of gene families.

The observation of relaxed purifying selection in SST-containing gene families might be a byproduct of SSTs tending to be only found in spiders; 17% of SST-containing *Latrodectus* & *Steatoda* gene clusters had no observable homolog to a nonspider UniProt protein (lineage-specific). This is a much higher percentage than non-SST *Latrodectus* & *Steatoda* gene clusters (4.6%), although similar to the *Latrodectus* & *Steatoda* gene clusters containing only low expression transcripts (15%). Indeed, we found that the *Latrodectus* & *Steatoda* gene families with lineage-specific transcripts have higher omega values than those gene families with nonspider homologs. However, SST-containing *Latrodectus* & *Steatoda* gene families consistently have higher omega values (mean omega = 0.22 for SST-containing, lineage-specific families; mean omega = 0.18 for non-SST lineage-specific families; mean omega = 0.12 for SST-containing families with nonspider homolog; mean omega = 0.07 for non-SST families with nonspider homolog). Analysis of variance supports the distinction between omega values depending on the lineage-specificity and whether the *Latrodectus* & *Steatoda* gene family contains an SST, but not an interaction between the two (fig. 8; supplementary table S4, Supplementary Material online).

Alternatively, the higher omega values in SST-containing families could be due to the fact that these families tend to have more paralogs than non-SST families (e.g., fig. 6). There is a weakly positive correlation between omega values and the number of additional genes for the SST-containing (*R*^2^ = 0.38, *P* = 0.007), and the non-SST (*R*^2^ = 0.03, *P* < 1e-15) *Latrodectus*, *Steatoda* & tick gene families (fig. 7*B*). The slopes of the linear models derived for each set are nearly identical (*b*_SST_ = 0.0039, *b*_non-SST_ = 0.0041; fig. 7*B*) but the intercept for the SST linear model is twice that of the non-SST linear model, suggesting that the difference in omega is inherent to the SSTs compared with the non-SSTs rather than simply a reflection of larger gene family sizes for SSTs.

## Discussion

Our high-quality multitissue transcriptomes for three cobweb weaving spider species greatly expand genomic resources for spiders and allow us to test hypotheses regarding the role of gene duplication in the evolution of genes expressed in spider silk glands. Phylogenetic and molecular distance analyses of thousands of gene families support the hypothesis that a large-scale gene duplication event occurred early in the diversification of spiders (fig. 5). As predicted, we found gene families containing transcripts specifically expressed in the silk glands of one of our cobweb weaving species—*L. hesperus* SSTs (Clarke et al. 2014)—were three times as likely to retain an ancient paralog pair than were generally expressed transcripts. We further found evidence for relaxed purifying selection in gene families with a greater number of retained paralog pairs. However, the SSTs showed consistently higher rates of amino acid replacement than generally expressed transcripts, even when controlling for gene family size.

A more nuanced understanding of the relationship between the ancient gene duplication event we inferred and the evolution of silk glands and fibers is dependent on successfully resolving its timing in relation to the origin of spider silk glands. Silk glands almost certainly evolved in the common ancestor of all spiders approximately 400 Ma (Selden et al. 2008; Foelix 2010; Starrett et al. 2012). We are less confident in the timing of the ancient duplication event, as it is difficult to accurately date and resolve large-scale duplication events of such antiquity, especially from distributions of substitution rates (Vanneste et al. 2013). The median molecular distance (d*S*) of older paralog pairs suggests the duplication event occurred around the same time as the divergence of the spider suborders, Mygalomorphae and Araneomorphae, approximately 380 Ma (Ayoub et al. 2007; Starrett et al. 2012; Wood et al. 2013). For comparison, the well-studied ancient whole-genome duplication event near the origin of the flower is closer to 160 Ma (Albert et al. 2013). The distribution of d*S* of the older spider paralog pairs (fig. 5*A*) and individual gene trees (fig. 5*B–D*) suggests that the large-scale duplication is even more ancient than the divergence of the spider suborders. The absence of numerous ancient paralogs within the scorpion, *M. martensii,* genome (Cao et al. 2013) supports the placement of the large-scale duplication event either exclusively within the spider lineage or in a subclade of arachnid orders including spiders but not scorpions (Regier et al. 2010; Bond et al. 2014; Fernández et al. 2014). Because our analysis did not include any Mesothelae representatives we are unable to confidently assign the older duplication event to the common ancestor of all living spiders.

If the ancient duplication event predates or coincides with the common ancestor of all living spiders, then it could have contributed to the origin of the silk spinning apparatus (e.g., spinnerets, silk glands, and fibers). It has been proposed that large-scale duplication events can have morphological consequences as the presence of four *Hox* clusters in vertebrates, which suggests two polyploidy events at their root, is associated with diversification of the vertebrate body plan (Ohno 1970; Holland et al. 1994). In the wandering spider, *C. salei*, duplicated *Hox* genes (*Ubx* 1 and 2) are both expressed in the abdominal limb buds that will eventually develop into spinnerets (Schwager et al. 2007). Both fossil and developmental genetic evidence suggest that spider spinnerets are homologs of abdominal appendages found on the fourth and fifth somites of horseshoe crabs, *Limulus*, which are basal chelicerates (Selden et al. 2008). Other arachnids do not develop homologs of these particular abdominal appendages, suggesting that the genes for appendage development were suppressed in the common ancestor of arachnids, but reactivated in spiders for spinneret formation (Selden et al. 2008). Reactivation might have been facilitated by a large-scale duplication event that included duplicating the *Hox* genes, allowing the evolution of the silk organs (e.g., spinnerets and possibly glands) and thus silk production in the spiders. If true, we predict that multiple older paralogs are coexpressed in the embryonic spinnerets and silk glands in spiders.

Even if the ancient duplication event occurred after the origin of the silk glands, it appears that it was instrumental in the evolution of transcripts with silk gland restricted expression. Black widow SSTs (with the exception of the spidroins) were almost exclusively paralogous with generally expressed transcripts, suggesting massive regulatory divergence after the duplication event. Additional functional specialization of SSTs is supported by their consistently higher amino acid replacement rates than other transcripts even when controlling for gene family size (fig. 7*B*). It is also likely that SSTs are coexpressed or physically interact with spidroins, which can result in correlated evolutionary rates as has been seen for *D. melanogaster* (Clark et al. 2012). The omega values calculated for the repetitive regions of spidroins within *Latrodectus* or within *Argiope* (garden spiders) range from 0.09 to 0.30 (Ayoub et al. 2013; Chaw et al. 2014), which are similar to or higher than the omega values we calculated for gene families containing other SSTs.

Two challenges to our hypotheses that a large-scale duplication event occurred early in the divergence of spiders and was important to the evolution of the silk glands is the fact that some gene families, including those directly involved with silk-production, like the spidroins, contain multiple duplication events and that some postdate the proposed ancient duplication event. The first outcome suggests that the bimodal distribution of d*S* for our paralog pairs (fig. 4) may derive from multiple independent duplication events with a high rate of paralog retention during a restricted time frame (discussed by Taylor et al. 2003). However, bimodal distributions of d*S* are almost exclusively associated with large-scale duplication events when complementary syntenic or karyotype evidence is available (see the extensive list in introduction to Vanneste et al. 2013). It is possible that highly saturated synonymous substitutions rates (d*S* > 2) could lead to incorrect inferences of the scale of the duplication event (Vanneste et al. 2013), but we restricted our paralog pairs to those with less saturation (d*S* < 2). Also, as multiple spidroin expansions postdate the divergence of Mygalomorphae and Araneomorphae (Garb et al. 2007; Ayoub and Hayashi 2008; Gaines and Marcotte 2008; Starrett et al. 2012), it is possible that paralogs deriving from ancient small scale duplication events might have contributed to the evolution of silk gland-specific transcripts. However, phylogenetic analyses of the spidroin gene family indicate a minimum of three paralogs present in the common ancestor of Mygalomorphae and Araneomorphae (Starrett et al. 2012), suggesting paralogs deriving from the ancient duplication event may have created early duplicates in the spidroin gene family.

In conclusion, we propose that a large-scale (e.g., whole genome or chromosomal) duplication event in spiders generated genetic material for the diversification of silk glands and fibers. Although sequencing complete spider genomes from each suborder should resolve the timing and scale of this event, our transcriptomes proved a powerful resource for identifying this large-scale duplication and demonstrating its importance in the evolution of tissue-specific transcripts. Unequal retention of paralogs in Araneomorphae and Mygalomorphae may have contributed to silk gland differentiation between the two suborders. However, our limited knowledge of the specific nonspidroins expressed in each specialized gland and their retention in different species currently restricts this analysis. Although the exact role that the large-scale duplication event played in the phenotypic and ecological diversification of spiders remains unknown, its placement prior to the divergence of the major spider suborders strongly suggests its importance in the ecological success of spiders.

## Supplementary Material

Supplementary tables S1–S4 and supplementary figures S1–S5 are available at *Genome Biology and Evolution* online (http://www.gbe.oxfordjournals.org/).

Supplementary Data
