# Supplementary material for: Spider Transcriptomes Identify Ancient Large-Scale Gene Duplication Event Potentially Important in Silk Gland Evolution
Source: Genome Biol Evol. 2015 Jun 8;7(7):1856–70. doi: 10.1093/gbe/evv110 (PMC4524477; doi:10.1093/gbe/evv110)
Supplement: Supplementary Data [file supp_7_7_1856__index.html]

Spider Transcriptomes Identify Ancient Large-Scale Gene Duplication Event Potentially Important in Silk Gland Evolution — Supplementary Data 

# Spider Transcriptomes Identify Ancient Large-Scale Gene Duplication Event Potentially Important in Silk Gland Evolution

## Supplementary Data

files

- Supplementary Data - pdf file
