# Supplementary material for: Spider Transcriptomes Identify Ancient Large-Scale Gene Duplication Event Potentially Important in Silk Gland Evolution
Source: Genome Biol Evol. 2015 Jun 8;7(7):1856–70. doi: 10.1093/gbe/evv110 (PMC4524477; doi:10.1093/gbe/evv110)
Supplement: Supplementary Data [file supp_evv110_SupplementaryMaterial_Clarke_et_al.pdf]

SUPPLEMENTARY FIGURES

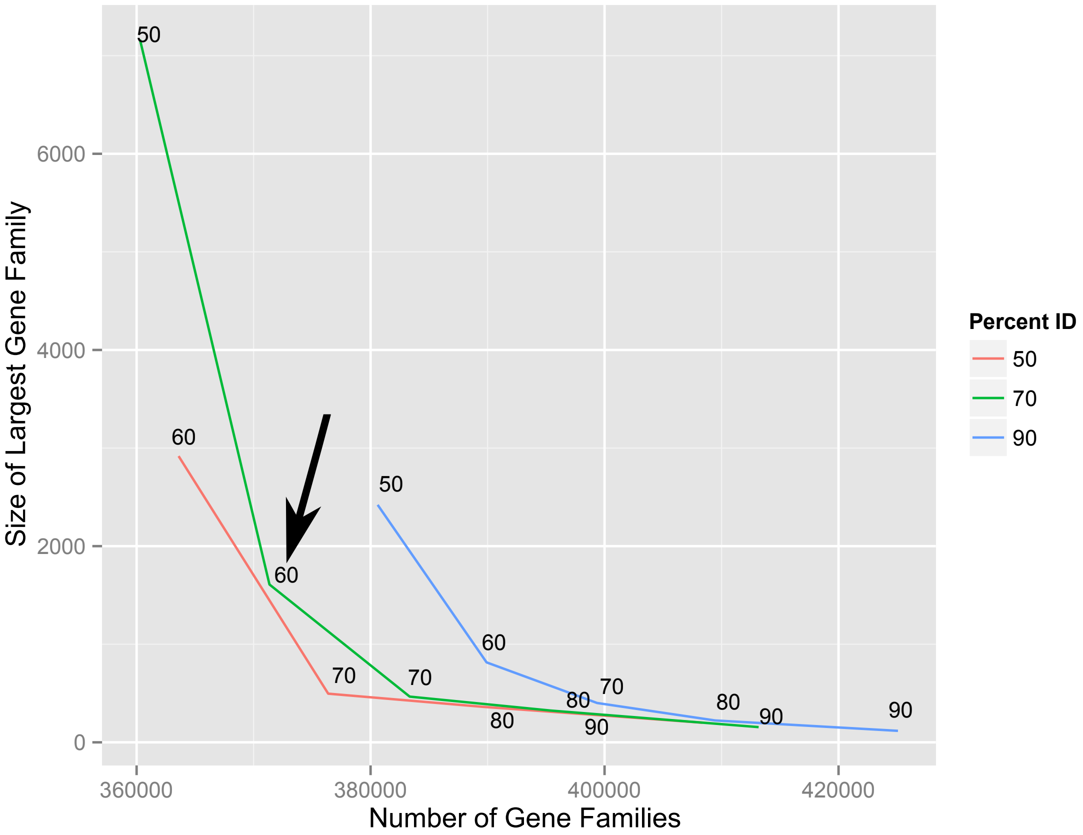

Figure S1. **Selection of BLASTCLUST parameters for optimal cluster size.** Clusters of transcripts that could represent gene families were constructed using different percent amino acid identity (ID, colored lines) and percent length coverage (labeled points) and analyzed based on the number of families (X-axis) and the size of the largest family (Y-axis). The goal was to group together homologous transcripts (minimize number of gene families) without combining non-homologous transcripts (minimize size of largest gene family). The parameters selected were 70% ID and 60% coverage (shown with arrow).

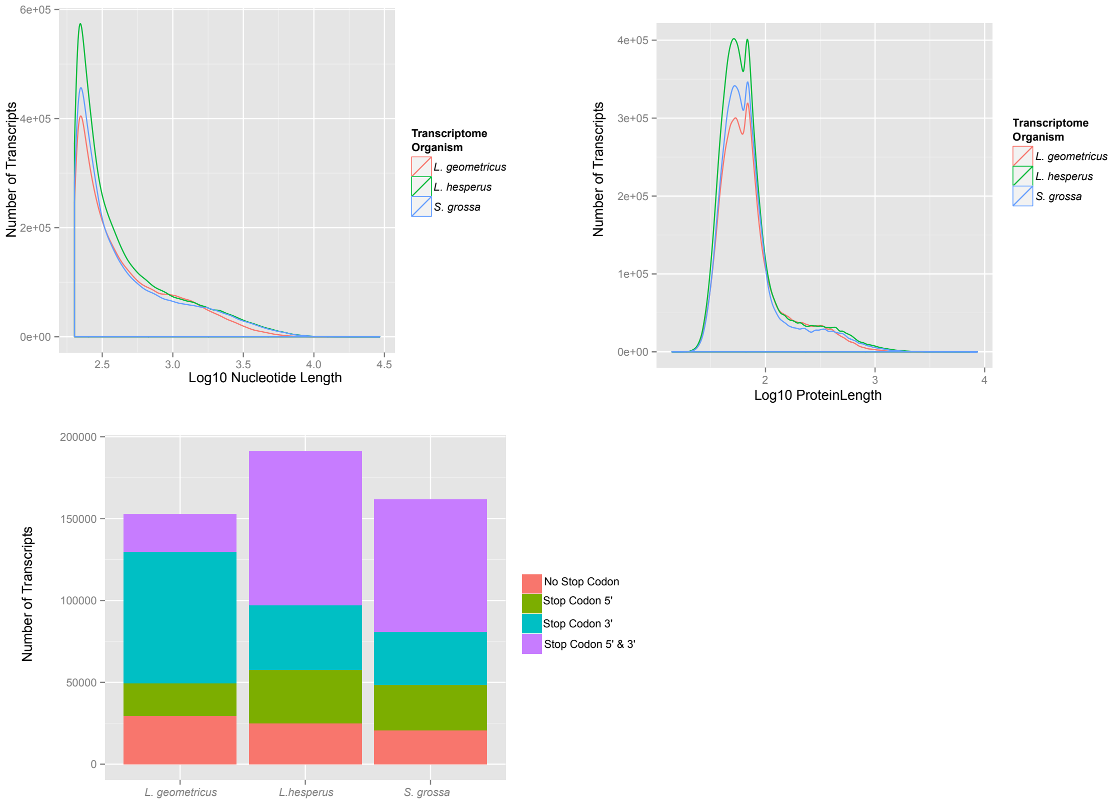

Figure S2. **Length distribution and completeness of transcripts and encoded proteins in multi-tissue transcriptomes of three cobweb weaving spider species.** The distributions of the nucleotide length (**A**) and the protein length (**B**) of transcripts are nearly identical for *Latrodectus hesperus, L. geometricus*, and *Steatoda grossa*. However, the percentage of transcripts whose longest open reading frames are bound by stop codons at the 5' and 3' ends (**C**) show that more of the *L. hesperus* and *S. grossa* transcripts contain complete coding sequences, while most of the *L. geometricus* transcripts are only 3' bound.

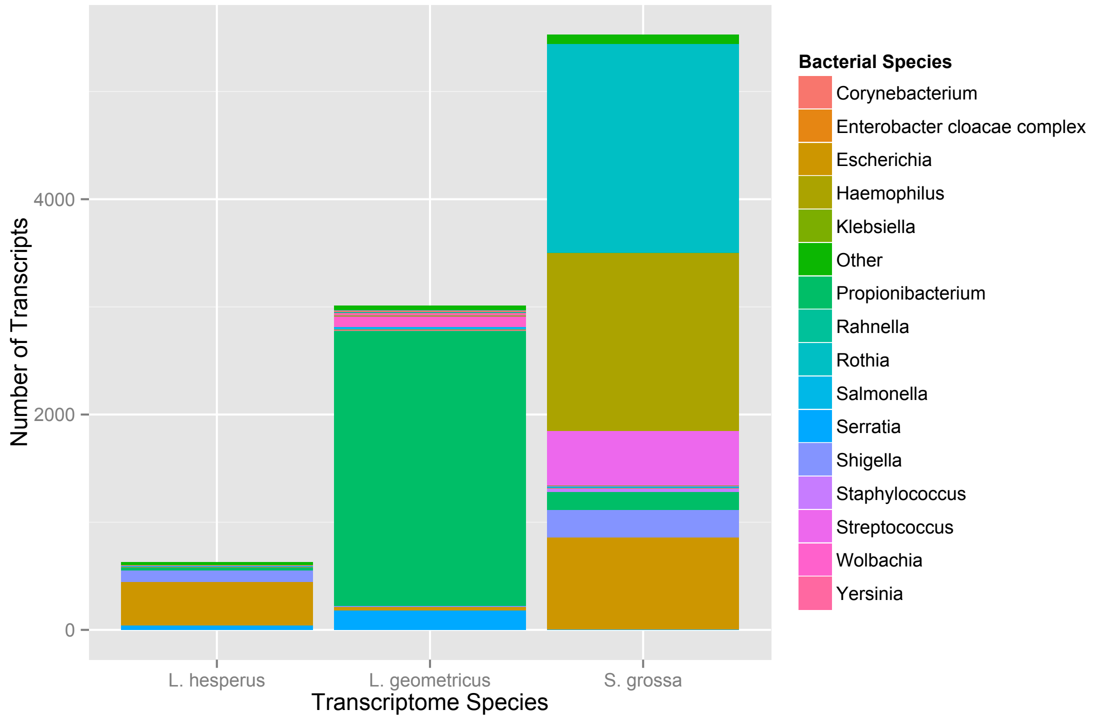

Figure S3. Bacterial sequences in the transcriptomes. The number of sequences with a best BLASTX match to a UniProt bacterial sequence at an e-value < 1e-50 and the bacterial genus associated with those sequences varies among draft transcriptomes of three cobweb weaving spider species. Bacterial genera with less than 10 transcripts were classified as “other”.

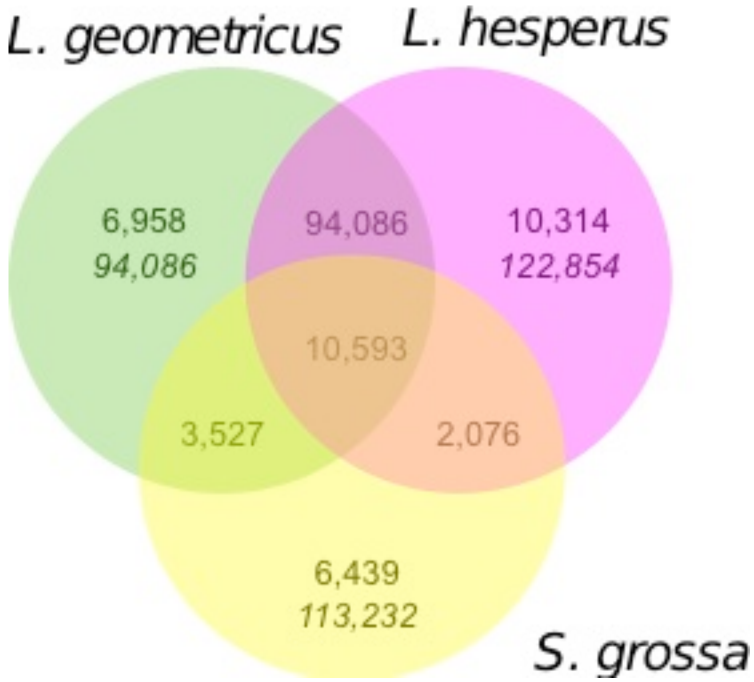

Figure S4. Gene cluster coverage in three cobweb weaving spider transcriptomes. The numbers of BLASTCLUST derived clusters with representative transcripts in the respective transcriptomes are shown in plain text. The numbers of single transcripts that did not cluster with any others are shown in italics.

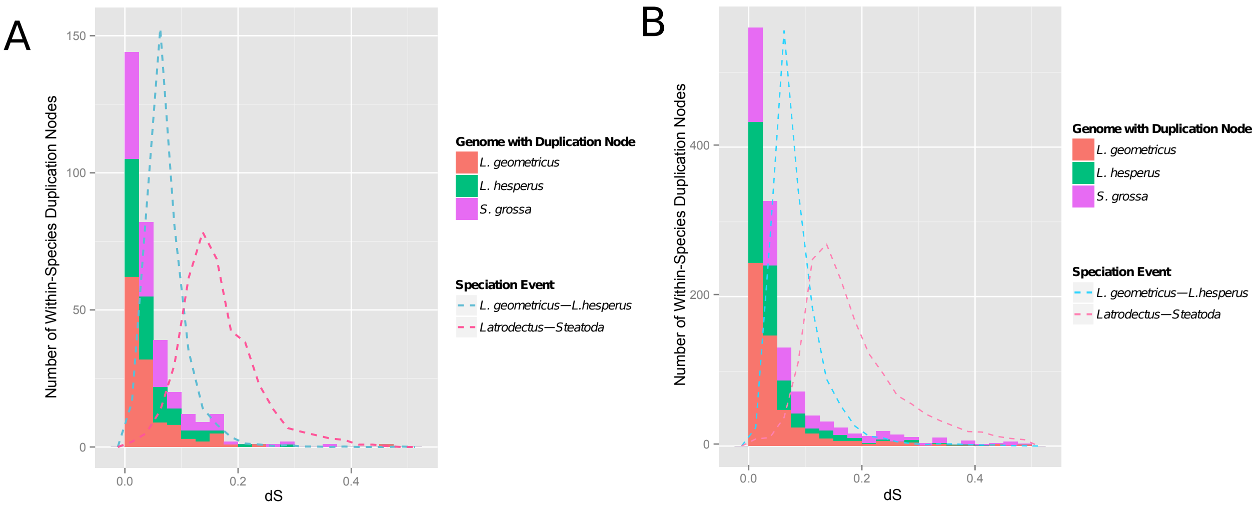

Figure S5. Duplication nodes occurring after cobweb weaving spider species' divergence have low molecular distances. Distributions of the mean number of synonymous substitutions per synonymous sites (dS) for paralog pairs descending from duplication nodes in the arachnid gene families (**A**) and in *Latrodectus* & *Steatoda* gene clusters (**B**) (see Figure 2 for delineation of gene families and clusters) that are exclusive to a single cobweb spider species genome show only a single peak at dS ~0.05. This distribution is similar to the younger duplication distribution in Figure 4. The dS distribution is also lower than the dS distributions of speciation events between cobweb weaving spider species as shown by the dotted lines.

SUPPLEMENTARY TABLES

Table S1. RNA-sequencing library information. RNA was isolated from 11-12 different tissue types from 1-29 individuals per species. Individuals were collected in August 2011, unless otherwise specified, and divided into two sets per species. Libraries generated at Johns Hopkins are shown under Library Type = JH. "Number of Paired End Reads" reflect number retained after removing low quality and rRNA sequences.

Species	Tissue	set of individuals	# individuals	Library Type	Number of Paired End Reads
L. hesperus	Aciniform	set 1+2+more	26	JH	14543268
L. hesperus	Aggregate	set 1	9	JH	10994145
L. hesperus	Aggregate	set 2	9	JH	9954507
L. hesperus	Cephalothorax	subset 1	1	JH	33572455
L. hesperus	Cephalothorax	collected July 2010	1	RNA-seq	50969807
L. hesperus	Flagelliform	set 1	9	JH	16273329
L. hesperus	Flagelliform	set 2	9	JH	16808798
L. hesperus	Major Ampullate	set 1	9	JH	6116968
L. hesperus	Major Ampullate	set 2	9	JH	9574596
L. hesperus	Male		1	JH	22730762
L. hesperus	Male		1	JH	18799106
L. hesperus	Minor Ampullate	set 1	9	JH	11679960
L. hesperus	Minor Ampullate	set 2	9	JH	11148437
L. hesperus	Ovary	subset 1	8	JH	6570003
L. hesperus	Ovary	set 2	9	JH	25729159
L. hesperus	Pyriform	subset 1 + 1 more	9	JH	12566537
L. hesperus	Pyriform	set 2	9	JH	15986335
L. hesperus	Tubuliform	subset 1	8	JH	18141905
L. hesperus	Tubuliform	subset 2	8	JH	16879141
L. hesperus	Venom	set 1	9	JH	15674162
L. hesperus	Venom	set 1	9	JH	24804712
L. hesperus	Venom	collected July 2010	7	RNA-seq	52044562
L. hesperus	Silk	collected Mar 2009	1	RNA-seq	15093424
S. grossa	Aciniform	set 1+2+more	29	JH	18079296
S. grossa	Aggregate	set 1	9	JH	21375473
S. grossa	Cephalothorax	subset 1	1	JH	28031599
S. grossa	Cephalothorax	collected July 2010	1	RNA-seq	58663915
S. grossa	Flagelliform	set 1	9	JH	21641167
S. grossa	Major Ampullate	set 1	9	JH	11385692
S. grossa	Major Ampullate	subset 2	11	JH	13837889
S. grossa	Male		1	JH	27842883
S. grossa	Male		1	JH	17003251
S. grossa	Minor Ampullate	set 1	9	JH	12244952

S. grossa	Minor Ampullate	subset 2	10	JH	15036559
S. grossa	Ovary	set 1	9	JH	21663292
S. grossa	Ovary	subset 2	11	JH	8974170
S. grossa	Pyriform	set 1 + subset 2	14	JH	23146750
S. grossa	Silk	collected July 2010	1	RNA-seq	79893301
S. grossa	Tubuliform	set 1	9	JH	25372714
S. grossa	Tubuliform	subset 2	11	JH	13252894
S. grossa	Venom	set 1	9	JH	14058967
S. grossa	Venom	set 2	14	JH	5047458
L. geometricus	Aciniform	subset 1 + 2	21	JH	15592765
L. geometricus	Aggregate	set 1	9	JH	10669428
L. geometricus	Aggregate	subset 2	10	JH	6829112
L. geometricus	Cephalothorax	subset 1	1	JH	11878029
L. geometricus	Flagelliform	set 1	9	JH	12723407
L. geometricus	Flagelliform	subset 2	10	JH	4219912
L. geometricus	Major Ampullate	set 1	9	JH	13978630
L. geometricus	Major Ampullate	subset 2	10	JH	13636997
L. geometricus	Male		4	JH	16098281
L. geometricus	Male		4	JH	15227691
L. geometricus	Minor Ampullate	set 1	9	JH	4832818
L. geometricus	Minor Ampullate	subset 2	10	JH	20640831
L. geometricus	Ovary	set 1	9	JH	17297978
L. geometricus	Ovary	subset 2	10	JH	12280306
L. geometricus	Tubuliform	subset 1	8	JH	17369795
L. geometricus	Tubuliform	subset 2	10	JH	10141938
L. geometricus	Venom	set 1	9	JH	17658481
L. geometricus	Venom	set 2	14	JH	6912185
L. geometricus	Silk	collected July 2010	1	RNA-seq	8101748
L. geometricus	Cephalothorax	collected July 2010	1	RNA-seq	46138872

Table S2: Means and likelihoods of the distributions of dS from the Gaussian mixture model of the *Latrodectus, Steatoda* & tick gene families. dS = mean number of synonymous substitutions per synonymous sites for paralog pairs descending from the duplication node.

Number of Distributions	2	3	4	5
Gaussian Mixture Model Means[1]	0.028	0.019	0.021	0.014
		0.056	0.061	0.03
				0.078
	0.533	0.568	0.506	0.51
			1.031	0.991
Log-Likelihood[2]	298.4551	377.7665	399.4224	451.4

1. Means ranked from lowest to highest with those suggestive of an ancient large-scale duplication event shaded in light grey. Means of distributions less than the putative large-scale duplication event are in dark grey, and means greater than the putative large -scale duplication event are in white.
2. Log-likelihood of the Gaussian mixtures modeling the dS distribution as returned by mixtools.

Table S3. Phylogenetic location of duplication nodes in 106 arachnid gene families that contained a scorpion and mygalomorph representative. Only duplication nodes with dS within the 95% range of dS values for the older Gaussian distribution (blue in Figure 4C) are shown.

Family ID	mean dS[1]	Phylogenetic Position of Duplication Node[2]	PP at Duplication Node[3]	PP of Children Node[4]	Phylogenetic Position at 50% PP[5]	Phylogenetic Position at 95% PP[6]
000008-0	0.88	Scorpion—Spider	Root	0.72;0.95;	Pre	Ambiguous
000008-0	0.16	Latrodectus—Steatoda	0.784	*;0.689;	Post	Ambiguous
000304-0	0.58	Scorpion—Spider	Root	0.538;0.655;	Pre	Ambiguous
000304-0	0.55	Scorpion—Spider	0.538	0.959;0.742;	Pre	Ambiguous
000422-0	0.84	Scorpion—Spider	Root	1.0;0.875;	Pre	Ambiguous
000422-0	0.45	Theridiidae—Eresidae	1	0.524;1.0;	Post	Post
000422-0	0.37	Araneomorph—Mygalomorph	1	1.0;0.875;	Intra	Intra
000620-0	0.43	Scorpion—Spider	Root	0.946;*;	Pre	Ambiguous
000620-0	0.53	Scorpion—Spider	0.946	0.795;0.691;	Pre	Ambiguous
000620-0	0.14	Theridiidae—Eresidae	0.901	0.742;*;	Post	Ambiguous
000620-0	0.15	Theridiidae—Eresidae	0.742	0.693;*;	Post	Ambiguous
000752-0	0.61	Scorpion—Spider	Root	*;1.0;	Pre	Ambiguous
000752-0	1.22	Scorpion—Spider	1	0.562;1.0;	Pre	Pre
000752-0	0.15	S. grossa	1	*;1.0;	Single Species	Single Species
000902-0	0.12	S. grossa	0.961	;;	Single Species	Ambiguous
000925-0	0.32	Araneomorph—Mygalomorph	0.584	0.671;*;	Intra	Ambiguous
000925-0	0.29	Araneomorph—Mygalomorph	0.671	0.865;*;	Intra	Ambiguous
001059-0	0.49	Araneomorph—Mygalomorph	Root	0.721;0.651;	Intra	Ambiguous
001059-0	0.42	Theridiidae—Eresidae	0.651	0.621;*;	Post	Ambiguous
001505-0	0.79	Araneomorph—Mygalomorph	Root	0.672;*;	Intra	Ambiguous
001697-0	1.05	Scorpion—Spider	Root	1.0;0.938;	Pre	Ambiguous
001697-0	0.92	Araneomorph—Mygalomorph	0.938	1.0;0.958;	Intra	Intra
001697-0	0.16	L. hesperus	1	;;	Single Species	Ambiguous
001697-2	0.86	Scorpion—Spider	Root	*;0.579;	Pre	Ambiguous
001697-2	0.93	Scorpion—Spider	0.579	0.995;0.797;	Pre	Ambiguous
001816-0	0.30	Araneomorph—Mygalomorph	0.733	NA	Polytomy	Polytomy
001866-0	0.33	Araneomorph—	0.958	0.999;0.996	Intra	Intra

		Mygalomorph		;		
002054-0	0.76	Scorpion—Spider	Root	0.744;0.944;	Pre	Ambiguous
002054-0	0.49	Araneomorph—Mygalomorph	0.978	0.543;1.0;	Intra	Intra
002267-0	0.32	Theridiidae—Eresidae	0.951	*;1.0;	Post	Post
002267-0	0.25	Theridiidae—Eresidae	1	1.0;1.0;	Post	Post
002356-0	1.21	Scorpion—Spider	0.66	NA	Polytomy	Polytomy
002356-0	0.95	Scorpion—Spider	0.684	NA	Polytomy	Polytomy
002356-0	0.98	Scorpion—Spider	0.813	1.0;0.836;	Pre	Ambiguous
002356-0	0.50	Latrodectus—Steatoda	1	0.964;1.0;	Post	Post
002356-0	0.69	Latrodectus—Steatoda	0.761	1.0;0.92;	Post	Ambiguous
002356-0	0.28	S. grossa	0.971	;;	Single Species	Ambiguous
002356-0	0.32	Latrodectus—Steatoda	0.92	0.794;0.558;	Post	Ambiguous
002356-0	0.19	S. grossa	0.758	1.0;*;	Single Species	Ambiguous
002396-0	0.45	Scorpion—Spider	Root	1.0;*;	Pre	Ambiguous
002396-0	0.53	Scorpion—Spider	1	0.944;1.0;	Pre	Pre
002467-0	0.42	Araneomorph—Mygalomorph	0.644	*;0.642;	Intra	Ambiguous
002527-0	0.83	Latrodectus—Steatoda	0.806	NA	Polytomy	Polytomy
002527-0	0.35	L. geometricus	0.618	;;	Single Species	Ambiguous
002527-0	0.08	S. grossa	0.666	;;	Single Species	Ambiguous
002630-0	0.63	Theridiidae—Eresidae	1	1.0;1.0;	Post	Post
002738-0	0.40	Araneomorph—Mygalomorph	1	0.998;0.67;	Intra	Intra
003133-0	0.52	Araneomorph—Mygalomorph	0.749	0.844;0.967;	Intra	Ambiguous
003133-0	0.21	S. grossa	0.676	NA	Polytomy	Polytomy
003141-0	1.00	Scorpion—Spider	Root	0.972;*;	Pre	Ambiguous
003141-0	0.19	Theridiidae—Gasteracantha	0.985	NA	Polytomy	Polytomy
003141-0	0.24	L. geometricus	0.505	;;	Single Species	Ambiguous
003259-0	0.38	Scorpion—Spider	Root	0.95;0.946;	Pre	Ambiguous
003259-0	0.17	L. hesperus—L. geometricus	0.95	*;1.0;	Post	Ambiguous
003259-0	0.30	Scorpion—Spider	0.946	1.0;*;	Pre	Ambiguous
003259-0	0.31	Scorpion—Spider	1	1.0;*;	Pre	Pre
003259-0	0.34	Scorpion—Spider	1	0.996;*;	Pre	Pre
003270-0	0.66	Scorpion—Spider	Root	0.651;0.955;	Pre	Ambiguous
003270-0	0.33	Araneomorph—	0.651	NA	Polytomy	Polytomy

		Mygalomorph				
003360-0	0.86	Scorpion—Spider	Root	0.833;0.907;	Pre	Ambiguous
003360-0	0.86	Scorpion—Spider	0.833	*;0.739;	Pre	Ambiguous
003360-0	1.11	Scorpion—Spider	0.739	0.621;0.627;	Pre	Ambiguous
003398-0	1.01	Scorpion—Spider	Root	1.0;0.833;	Pre	Ambiguous
003398-0	0.27	Latrodectus—Steatoda	1	1.0;*;	Post	Post
003398-1	0.58	Araneomorph—Mygalomorph	0.774	1.0;1.0;	Intra	Intra
003398-1	0.33	Araneomorph—Mygalomorph	1	1.0;0.984;	Intra	Intra
003738-0	0.45	Scorpion—Spider	0.532	NA	Polytomy	
003738-0	0.45	Scorpion—Spider	0.648	NA	Polytomy	Polytomy
003738-0	0.63	Scorpion—Spider	0.675	0.753;0.837;	Pre	Ambiguous
003738-0	0.78	Scorpion—Spider	0.837	1.0;0.798;	Pre	Ambiguous
003738-0	0.79	Scorpion—Spider	0.661	0.97;0.931;	Pre	Ambiguous
003738-0	0.63	Latrodectus—Steatoda	0.97	0.998;0.991;	Post	Post
003819-0	0.46	Scorpion—Spider	Root	*;0.962;	Pre	Ambiguous
003819-0	0.45	Scorpion—Spider	0.962	*;0.897;	Pre	Ambiguous
003819-0	0.52	Scorpion—Spider	0.897	1.0;1.0;	Pre	Pre
004057-0	0.12	S. grossa	0.953	;;	Single Species	Ambiguous
004057-0	0.12	L. hesperus	0.99	;;	Single Species	Ambiguous
004124-0	0.67	Scorpion—Spider	Root	0.706;0.994;	Pre	Ambiguous
004124-0	0.17	Theridiidae—Eresidae	1	1.0;1.0;	Post	Post
004204-0	0.62	Scorpion—Spider	Root	0.954;0.567;	Pre	Ambiguous
004204-0	0.13	Theridiidae—Eresidae	0.883	0.999;0.889;	Post	Ambiguous
004460-0	0.47	Scorpion—Spider	1	0.972;0.973;	Pre	Pre
004460-0	0.65	Scorpion—Spider	0.972	0.583;0.529;	Pre	Ambiguous
004460-0	0.32	Latrodectus—Steatoda	0.773	0.871;0.918;	Post	Ambiguous
004460-0	0.13	L. geometricus	1	;;	Single Species	Ambiguous
004817-0	0.60	Araneomorph—Mygalomorph	0.903	0.833;1.0;	Intra	Ambiguous
004817-0	0.24	Theridiidae—Gasteracantha	0.676	0.505;*;	Post	Ambiguous
004817-0	0.15	L. geometricus	0.736	0.747;*;	Single Species	Ambiguous

004817-0	0.29	L. geometricus	0.747	;;	Single Species	Ambiguous
004817-0	0.19	Araneomorph—Mygalomorph	1	0.78;*;	Intra	Ambiguous
004867-0	0.22	L. geometricus	0.98	;;	Single Species	Ambiguous
004875-0	0.80	Araneomorph—Mygalomorph	0.713	0.926;1.0;	Intra	Ambiguous
004875-0	0.70	Theridiidae—Eresidae	1	1.0;1.0;	Post	Post
004940-0	0.39	Araneomorph—Mygalomorph	0.596	0.871;*;	Intra	Ambiguous
004943-0	0.46	Scorpion—Spider	Root	*;0.744;	Pre	Ambiguous
004943-0	0.53	Scorpion—Spider	0.744	0.576;0.757;	Pre	Ambiguous
004943-0	0.31	Latrodectus—Steatoda	0.999	1.0;0.95;	Post	Post
005011-0	0.09	S. grossa	0.997	;;	Single Species	Ambiguous
005011-0	0.11	L. hesperus	0.992	;;	Single Species	Ambiguous
005198-0	0.13	L. hesperus—L. geometricus	0.998	0.546;*;	Post	Ambiguous
005198-0	0.37	S. grossa	0.974	;;	Single Species	Ambiguous
005779-0	0.62	Scorpion—Spider	Root	1.0;1.0;	Pre	Pre
005779-0	0.40	Theridiidae—Eresidae	0.938	*;1.0;	Post	Ambiguous
005779-0	0.17	S. grossa	1	;;	Single Species	Ambiguous
005876-0	0.21	Ther	0.994	0.778;1.0;	Post	Post
006382-1	0.85	Scorpion—Spider	Root	0.974;0.533;	Pre	Ambiguous
006382-1	0.71	Scorpion—Spider	0.974	*;0.813;	Pre	Ambiguous
006766-0	0.31	Araneomorph—Mygalomorph	0.615	0.997;1.0;	Intra	Intra
007235-0	0.26	Scorpion—Spider	0.785	0.782;0.957;	Pre	Ambiguous
007420-0	0.64	Scorpion—Spider	Root	0.641;0.612;	Pre	Ambiguous
007420-0	0.17	L. hesperus—L. geometricus	0.877	0.913;*;	Post	Ambiguous
007426-0	0.23	S. grossa	0.998	;;	Single Species	Ambiguous
007452-0	0.60	Scorpion—Spider	Root	0.913;1.0;	Pre	Ambiguous
007452-0	0.62	Araneomorph—Mygalomorph	0.984	1.0;1.0;	Intra	Intra
007728-0	0.48	Scorpion—Spider	Root	1.0;1.0;	Pre	Pre
007728-0	0.47	Scorpion—Spider	1	0.987;0.59;	Pre	Pre
007728-0	0.27	Latrodectus—Steatoda	0.987	0.974;*;	Post	Post
007728-0	0.11	L. geometricus	1	;;	Single Species	Ambiguous

007896-0	0.84	Scorpion—Spider	Root	0.747;1.0;	Pre	Ambiguous
008054-0	0.48	Scorpion—Spider	Root	0.912;*;	Pre	Ambiguous
008054-0	0.41	Scorpion—Spider	0.912	0.976;0.666;	Pre	Ambiguous
008054-0	0.36	Theridiidae—Eresidae	0.751	1.0;0.558;	Post	Ambiguous
008486-0	0.44	Araneomorph—Mygalomorph	0.932	0.977;0.945;	Intra	Ambiguous
008486-0	0.57	Theridiidae—Eresidae	0.945	*;1.0;	Post	Ambiguous
009036-0	0.69	Latrodectus—Steatoda	0.682	1.0;1.0;	Post	Post
009036-0	0.64	Latrodectus—Steatoda	1	0.797;1.0;	Post	Post
009036-0	0.62	L. hesperus—L. geometricus	0.797	NA	Polytomy	Polytomy
009036-0	0.52	L. hesperus—L. geometricus	0.626	1.0;1.0;	Post	Post
009036-0	0.22	L. hesperus—L. geometricus	0.929	0.947;1.0;	Post	Ambiguous
009277-0	0.81	Scorpion—Spider	1	*;0.979;	Pre	Pre
009277-0	0.84	Scorpion—Spider	0.979	0.991;0.997;	Pre	Pre
009277-0	1.20	Scorpion—Spider	0.991	1.0;0.991;	Pre	Pre
009277-0	0.67	Latrodectus—Steatoda	1	1.0;0.694;	Post	Post
009277-0	0.82	Latrodectus—Steatoda	0.694	0.592;1.0;	Post	Ambiguous
009277-0	0.82	Latrodectus—Steatoda	0.592	1.0;*;	Post	Ambiguous
009277-0	0.75	Latrodectus—Steatoda	1	1.0;1.0;	Post	Post
009277-0	0.46	Latrodectus—Steatoda	1	0.999;*;	Post	Post
009277-0	0.44	Latrodectus—Steatoda	1	0.996;1.0;	Post	Post
009277-0	0.93	Scorpion—Spider	0.991	1.0;0.804;	Pre	Pre
009277-0	0.11	L. hesperus	0.986	NA	Polytomy	Polytomy
009317-0	0.62	Theridiidae—Eresidae	0.607	*;0.703;	Post	Ambiguous
009481-0	0.63	Scorpion—Spider	0.717	0.792;*;	Pre	Ambiguous
009481-0	0.23	Latrodectus—Steatoda	0.98	0.879;*;	Post	Ambiguous
009486-0	0.83	Scorpion—Spider	Root	1.0;0.974;	Pre	Pre
009486-0	0.75	Araneomorph—Mygalomorph	0.962	1.0;0.987;	Intra	Intra
009486-0	0.36	Theridiidae—Eresidae	0.744	0.91;1.0;	Post	Ambiguous
009576-0	0.55	Araneomorph—Mygalomorph	0.525	1.0;0.996;	Intra	Intra
009576-0	0.46	Araneomorph—Mygalomorph	1	1.0;0.908;	Intra	Intra
009576-0	0.35	Araneomorph—Mygalomorph	0.996	*;0.674;	Intra	Ambiguous
009577-0	0.44	Latrodectus—Steatoda	0.934	*;0.768;	Post	Ambiguous
009577-0	0.45	Latrodectus—Steatoda	0.768	0.96;0.783;	Post	Ambiguous
009577-0	0.19	Latrodectus—Steatoda	0.96	0.96;*;	Post	Post
009990-0	0.09	L. geometricus	0.992	;;	Single Species	Ambiguous

010259-0	0.36	Latrodectus—Steatoda	1	*;0.616;	Post	Ambiguous
010259-0	0.70	Latrodectus—Steatoda	0.616	1.0;0.562;	Post	Ambiguous
010259-0	0.16	Latrodectus—Steatoda	1	0.646;*;	Post	Ambiguous
010265-0	0.41	Scorpion—Spider	0.944	1.0;1.0;	Pre	Pre
010265-0	0.20	S. grossa	1	1.0;*;	Single Species	Single Species
010284-0	0.63	Scorpion—Spider	1	0.964;0.766;	Pre	Pre
010284-0	0.17	Latrodectus—Steatoda	1	NA	Polytomy	Polytomy
010310-0	0.66	Ther	0.748	0.959;1.0;	Post	Post
010310-0	0.41	Latrodectus—Steatoda	0.959	0.996;1.0;	Post	Post
010310-0	0.10	L. geometricus	1	;;	Single Species	Ambiguous
010683-0	0.44	Scorpion—Spider	Root	1.0;0.688;	Pre	Ambiguous
010683-0	0.67	Scorpion—Spider	1	*;1.0;	Pre	Pre
010800-0	0.61	Araneomorph—Mygalomorph	0.846	1.0;0.633;	Intra	Ambiguous
011058-0	0.56	Theridiidae—Eresidae	1	0.952;1.0;	Post	Post
011065-0	0.30	Scorpion—Spider	0.571	0.596;*;	Pre	Ambiguous
011065-0	0.10	L. geometricus	0.847	;;	Single Species	Ambiguous
011139-0	0.60	Araneomorph—Mygalomorph	0.832	0.867;0.868;	Intra	Ambiguous
011139-0	0.95	Theridiidae—Eresidae	0.868	*;0.897;	Post	Ambiguous
011181-0	0.53	Araneomorph—Mygalomorph	0.558	0.742;0.567;	Intra	Ambiguous
011259-0	0.58	Araneomorph—Mygalomorph	1	1.0;0.506;	Intra	Intra
011798-0	0.43	Theridiidae—Eresidae	0.879	0.534;0.998;	Post	Ambiguous
011798-0	0.14	Latrodectus—Steatoda	0.998	0.701;*;	Post	Ambiguous
011983-0	0.44	Scorpion—Spider	Root	0.984;*;	Pre	Ambiguous
011983-0	0.44	Scorpion—Spider	0.984	*;0.903;	Pre	Ambiguous
011983-0	0.54	Scorpion—Spider	0.903	1.0;0.984;	Pre	Pre
011983-0	0.30	Araneomorph—Mygalomorph	1	*;0.79;	Intra	Ambiguous
012085-0	0.23	S. grossa	1	;;	Single Species	Ambiguous
012581-0	0.48	Araneomorph—Mygalomorph	0.964	1.0;1.0;	Intra	Intra
014158-0	0.52	Scorpion—Spider	Root	*;1.0;	Pre	Ambiguous
014158-0	0.35	Araneomorph—Mygalomorph	0.708	0.938;0.792;	Intra	Ambiguous
014158-0	0.20	Theridiidae—Eresidae	0.792	0.917;1.0;	Post	Ambiguous
014393-0	0.78	Scorpion—Spider	Root	0.989;1.0;	Pre	Pre
014393-0	0.23	Araneomorph—Mygalomorph	1	0.995;*;	Intra	Intra

014909-0	0.71	Scorpion—Spider	Root	0.608;0.741;	Pre	Ambiguous
014909-0	0.46	Araneomorph—Mygalomorph	0.741	0.962;*;	Intra	Ambiguous
015306-0	0.27	S. grossa	0.947	NA	Polytomy	Polytomy
015312-0	0.29	Araneomorph—Mygalomorph	0.811	0.8;0.997;	Intra	Ambiguous
015757-0	0.16	S. grossa	0.921	NA	Polytomy	Polytomy
016752-0	0.53	Araneomorph—Mygalomorph	0.633	0.829;0.829;	Intra	Ambiguous
016752-0	0.13	S. grossa	0.988	;;	Single Species	Ambiguous
016989-0	0.60	Araneomorph—Mygalomorph	0.732	*;0.531;	Intra	Ambiguous
017095-0	0.28	Araneomorph—Mygalomorph	1	0.955;0.789;	Intra	Intra
018355-0	0.65	Araneomorph—Mygalomorph	0.934	*;0.609;	Intra	Ambiguous
018355-0	0.37	Araneomorph—Mygalomorph	0.609	0.554;0.837;	Intra	Ambiguous
019266-0	0.51	Scorpion—Spider	Root	1.0;*;	Pre	Ambiguous
019266-0	0.60	Scorpion—Spider	1	0.992;0.636;	Pre	Pre
019266-0	0.57	Scorpion—Spider	0.992	*;0.583;	Pre	Ambiguous
019266-0	0.20	Araneomorph—Mygalomorph	1	*;0.55;	Intra	Ambiguous
022021-0	0.59	Theridiidae—Eresidae	0.988	1.0;0.796;	Post	Post
022021-0	0.25	L. hesperus	0.993	;;	Single Species	Ambiguous
022021-0	0.16	S. grossa	0.941	0.888;*;	Single Species	Ambiguous
022911-0	0.38	Theridiidae—Eresidae	0.802	NA	Polytomy	Polytomy
022911-0	0.21	S. grossa	0.863	;;	Single Species	Ambiguous
031617-0	0.28	Araneomorph—Mygalomorph	0.678	0.947;*;	Intra	Ambiguous

1. Mean pairwise dS of all descendant gene pairs on opposite sides of the duplication node.
2. The oldest speciation event that occurred after the duplication node as identified using Notung species tree–gene tree reconciliation and rooting.
3. Mr. Bayes calculated posterior probabilities (PP) for the duplication node. Root indicates that the node was the root in the Mr. Bayes returned tree, and lacks a posterior probability.
4. Mr. Bayes calculated posterior probabilities (PP) for the two branches that are children to the duplication nodes where available, separated by a semi-colon. Asterisks indicate that child is a leaf node and lacks a posterior probability. In cases of an unresolved polytomy, no posterior probabilities are given.
5. Indicates location of the duplication node if the duplication node and both of the children nodes have PP ≥ 0.50 or is a leaf. “Pre” indicates the duplication node occurred before

the divergence between scorpions and spiders; “intra” duplication nodes occurred between the divergence of scorpions and spiders and the divergence of aranaeomorphs and mygalomorphs; and “post” duplication nodes follow the divergence of aranaeomorphs and mygalomorphs. Also shown are those duplication nodes that are ambiguous, polytomic, or located exclusively in a single genome.

6. Same as 5, except that the duplication node and at least one of the children nodes have PP ≥ 0.95.

Table S4. Multi-ANOVA for omega in *Latrodectus & Steatoda* gene clusters gauging lineage-specificity (LS) and silk specific transcripts (SSTs).

	df	Sum Sq	Mean Sq	F-Value	Pr(>F)
LS[1]	1	3.87	3.875	339.288	<2e-16
SST[2]	1	0.30	0.300	26.250	3.08e-07
LS:SST	1	0	0.000	0.024	0.876
Residuals	7264	82.96	0.011		

1. Binary variable for *Latrodectus & Steatoda* gene clusters with no observed homolog to a non-spider protein in UniProt as determined by BLASTX (e-value < 1e-5).
2. Binary variable for *Latrodectus & Steatoda* gene clusters where at least one *L. hesperus* sequence is a best BLASTN (e-value < 1e-50) to a SST in Clarke et al (2014).
